# Veterinary Ethics3Read before the Kansas City Veterinary College Association, Kansas City, Mo., November 26, 1898.

**Published:** 1899-02

**Authors:** Joseph W. Parker

**Affiliations:** Student, Kansas City Veterinary College


### VETERINARY ETHICS.[3]

By Joseph W. Parker,

STUDENT, KANSAS CITY VETERINARY COLLEGE.

Ethics is a standard of morals applied to the conditions of life. The word is used synonymously with duty, right, morality. There are certain ethical precepts that are universally agreed to: that murder, malice, and theft are wrong; that each person is under obligations to respect personal rights of others; that each is entitled to enjoy the benefits of his own labor without interference or molestation.

[1] 14 urine. [2] Died after second 2.0.

[3] Read before the Kansas City Veterinary College Association, Kansas City, Mo., November 26, 1898.

Attempts have been made to formulate a code of ethics that shall express the ideas of right and wrong on which society is builded. Such a code is and must be incomplete, for each individual has his own ethical code, which is as distinct from others as his own personality. The inability to construct a universal code makes necessary a division of the subject into general and private ethics. The former consists of those principles which are universally recognized as correct morals. Popular customs, and both common and written law, take notice, are founded on these universally accepted ethical precepts. Private ethics apply to those conditions where there is difference of practice. Still another division of the so-called science is special ethics, which apply only to certain classes of people.

Every ethical code, whether special, personal, or general, springs from the principles of general ethics, and is but an interpretation of these general principles adapted to specific conditions. The followers of every occupation and calling have their special ethical ideas, which are, in some instances, perversions of right. For instance: The huckster sees no wrong in placing the best apples on the top of the measure; the lawyer feels that he does right in defending a criminal, no matter what the crime; the newspaper writer colors his articles to agree with the policy of the paper for which he writes, even though his own opinions are just the opposite; the conscience of the politician is proverbial. Like distinguishing between animal and vegetable life, we may usually be able to determine the one from the other at a glance, but occasionally conditions are presented where it is difficult to say which is the wrong.

We may not with arrogance fix a measure of morality and require every other to fill and conform to that measure with mathematical exactness; but, in so far as agreement is practically unanimous, an ethical code may be formulated with safety under which human actions will be judged and the approval of society or its reprobation be the meed impelling to duty.

Veterinary ethics applies to the conduct of veterinarians as related to each other in professional capacity. Associations of veterinarians, notably the United States Veterinary Medical Association, in order to promote the coöperation of each for the good of all, have formulated certain rules of conduct, which in specific manner relate to the following general and universally accepted principles:

1. Honesty is the first requirement of honor.

2. Every service should receive a just reward.

3. Every man deserves consideration on his merits, not on his pretenses; nor should the public be prejudiced by false representations of others.

4. Proper courtesy should be observed in all intercourse.

The letter of the adopted code is necessarily incomplete as a guide to honest and honorable conduct. That is, it is possible that a practitioner may transgress the spirit of ethics, may resort to unmanly and dishonorable expedients to get or to retain practice or prestige without actually being liable to ostracism for transgression of veterinary ethics as expressed in the code. Hence there are both written and unwritten veterinary ethics. It is the latter, especially, the foundation of every code, that should interest us as students.

From the general principles above stated the following thoughts are derived:

Since each service should have a just reward, cutting of prices to figures that are not remunerative is dishonorable, for it does not benefit him who gets the practice, and its only object is to break up the practice of a competitor. Moreover, cheap work does not benefit the patron in the long run, for it means poor service, and that is dear at any price.

Honesty is always a virtue. No honorable man will pretend to higher attainments than he possesses by laying claim to a title to which he has no right. If there is any advantage in affixing D.V.S. or D.V.M. to the name, that advantage is the vested property of him who has earned the title by the necessary study and labor to qualify himself for the pursuit of his calling. To falsely assume such a title, while not a crime in some States, is nevertheless theft. A quack brings discredit on the profession, if he be discovered or not. It is only by holding high ideals of honor that we may be enabled to respect ourselves and win the esteem of the public.

Along this line of thought, the foisting of specific remedies on the market, to be administered by laymen, is dishonest, for we well know that the remedy must be specially adapted to each case, and that no layman is competent to judge when that specific remedy is indicated. Much less is it honorable to gull the public with cure-alls—Giles' lotions for every ill that animal flesh is heir to. Especially should regular veterinarians avoid such means of making money. A quack, by dishonorable means, imposing on the ignorance of laymen, can pass a parasitic existence, getting his living from whom he renders no valuable service. The dignity and utility of veterinary science demand that its followers obtain the respect and confidence of the public by conscientiously rendering valuable service, and it is only by these means that the profession will be advanced in the esteem of the public to that high standing which its utility merits.

One method of the itinerant "hoss-doctor" (who, fortunately, is disappearing before the regular veterinarian) was to advertise his coming in local papers, handbills, posters, etc. To announce his wonderful abilities, and then for a few days impose on the ignorance of stock-owners, and, perhaps, by sharp tricks in trading augment his unearned income, and, having gone through the pockets of the long line of suckers every ready to be gulled, seek new fields for the pursuit of dishonorable practices. The merest tyro in professional learning and practical ability, if he posess the mental and moral requisites for a fakir, can make financial success of such a business, but he is sure to bring reproach upon the calling he unworthily follows. For this reason advertising of any character, beyond proper business cards, is tabooed by reputable veterinarians.

It is needless to state that any attempt to build up a practice by means calculated to bring a reputable competitor into reproach is dishonorable, and, in the end, impolitic. But I go further with the statement: It is neither right nor politic for a veterinarian to busy himself in injuring the business of another, even if the methods of the competitor are in violation of the ethical code. I do not mean that he should gloss over the ignorance or trickery of his competitor, but that he should maintain discreet silence in such matters. An intelligent public will by and by detect the impostor and award its patronage to the conscientious, capable veterinarian; whereas, should a practitioner expose the infamy even of a quack, and thereby become embroiled in animadverse controversy, reproach would fall upon both and on the profession generally. He owes it to the profession, therefore, to solicit patronage only by open and fair means and on his merits as a veterinarian. Obtaining practice through bribes to servants, subordinates, jockeys, and stablemen is also unprofessional and reprehensible, though a frequent resort of some veterinarians.

I am unprepared to discuss contract work, either for livestock insurance companies or for corporations and companies generally. If the service rendered receives fair remuneration, I see no reason why such contracts should be condemned; but there is a great temptation to do such work at very low rates for the benefit of the advertising it gives, thereby lowering prices generally.

In all consultations, after examining the patient, the veterinarians should withdraw to some private place and discuss the case, the attending veterinarian having first given all possible information as to the history of the case and previous treatment. The consulting veterinarian will then state his diagnosis and the treatment he would advise. If there be disagreement on any point, each will give respectful consideration to the views of the other. Having, if possible, arrived at agreement, it is the duty of the attending veterinarian to announce to the owner, in the presence of the other, the result of the consultation, and, if there be disagreement, either adopt the treatment suggested by the consulting veterinarian or to politely tender his severance from the case. Unless such severance should be accepted by the owner, however, and the consulting veterinarian then and there given charge of the case, he is not privileged to revisit it unless again called in consultation or at the request of the attending veterinarian. In all questions of ethics he who has constantly in mind the requirements of courtesy and the demands of justice cannot err greatly in his duty.

Unlike the fraternity of human practitioners, veterinarians have not a history of thousands of years on which to base their ethics, and are not encumbered with precedents established in the dark ages. The treatment of domestic animals by men specially qualified is in its infancy, especially in America. In the West and South there are more non-graduates than graduated veterinarians in the field. Quacks abound.

The public judges veterinary science by its representatives, whose average qualifications are none too high and whose sense of honor none too acute. Legislation is notoriously deficient. Little fellowship can obtain between veterinarians and the practising non-graduates, for obvious reasons. In the sparsely occupied territory thorough organization and effective coöperation are well-nigh impossible. Circumstances must, to a great extent, determine the practice of veterinary ethics.

The increasing numbers of regular graduates, the extension of veterinary organizations, and the growing urgency of more intelligent care of domestic animals, both from financial and sanitary standpoints, portend the disappearance of non-graduates. Favorable State and municipal legislation is in prospect. A fuller recognition of the utility and dignity of the science is foreshadowed. The realization of these desirable conditions is dependent on veterinarians.

